# An exploration of crowdsourcing citation screening for systematic reviews

**DOI:** 10.1002/jrsm.1252

**Published:** 2017-07-04

**Authors:** Michael L. Mortensen, Gaelen P. Adam, Thomas A. Trikalinos, Tim Kraska, Byron C. Wallace

**Affiliations:** ^1^ Netcompany A/S Aarhus C Denmark; ^2^ Health Services, Policy and Practice Brown University Providence RI USA; ^3^ Computer Science Brown University Providence RI USA; ^4^ College of Computer and Information Science Northeastern University Boston MA USA

**Keywords:** crowdsourcing (MeSH), evidence‐based medicine (MeSH), review literature as topic (MeSH), study selection, systematic review methods

## Abstract

Systematic reviews are increasingly used to inform health care decisions, but are expensive to produce. We explore the use of *crowdsourcing* (distributing tasks to untrained workers via the web) to reduce the cost of screening citations. We used Amazon Mechanical Turk as our platform and 4 previously conducted systematic reviews as examples. For each citation, workers answered 4 or 5 questions that were equivalent to the eligibility criteria. We aggregated responses from multiple workers into an overall decision to include or exclude the citation using 1 of 9 algorithms and compared the performance of these algorithms to the corresponding decisions of trained experts. The most inclusive algorithm (designating a citation as relevant if *any* worker did) identified 95% to 99% of the citations that were ultimately included in the reviews while excluding 68% to 82% of irrelevant citations. Other algorithms increased the fraction of irrelevant articles excluded at some cost to the inclusion of relevant studies. Crowdworkers completed screening in 4 to 17 days, costing $460 to $2220, a cost reduction of up to 88% compared to trained experts. Crowdsourcing may represent a useful approach to reducing the cost of identifying literature for systematic reviews.

## 1 BACKGROUND AND SIGNIFICANCE

Systematic and scoping reviews synthesize the available relevant evidence on a topic. These reviews inform all levels of decision making about health, from personal decisions to policy‐making. However, conducting systematic reviews is laborious and hence expensive: producing a single review can require thousands of person‐hours.^1^ The exponential expansion of the biomedical literature base has imposed an increased burden on reviewers who have to screen more citations to find relevant articles, thus multiplying costs. Researchers can no longer keep up with the primary literature, and this hinders the practice of evidence‐based care.^2^ This has motivated interest in methods to modernize certain aspects of the systematic review process (eg, via automation).^3^, ^4^, ^5^

Citation screening is the tedious yet critical step of winnowing down the large set of citations retrieved via a broad database query to those eligible for inclusion in a systematic review. Typically, this involves screening thousands of citations (titles, abstracts, and keywords) to identify the small subset of potentially eligible studies to be considered further for inclusion. Citations screened in at this phase are subsequently evaluated in full text. Methods for semiautomating this step using data mining have been proposed as a potential means of reducing the workload.^3^

## 2 OBJECTIVE

In this article, we investigate the potential of *crowdsourcing* to reduce the workload involved in citation screening for systematic reviews. We refer to crowdsourcing as relying on a group of individuals to complete “microtasks” (usually via the Internet) that are perhaps too difficult for a computer to accomplish with current artificial intelligence methods. Amazon (creator and owner of the Mechanical Turk crowdsourcing platform) refers to this as “artificial artificial intelligence.”

In our experiments, we used citation data and screening decisions from 4 previously conducted systematic reviews. We hired crowdworkers to make screening decisions for citations after they had been given a brief explanation of the task and criteria. Workers on Mechanical Turk are unlikely to have any prior experience with or knowledge of evidence‐based medicine. Despite this lack of familiarity, we found that crowdworkers had relatively high‐screening accuracy, demonstrating the potential of crowdsourcing to facilitate evidence‐based medicine. Ultimately, we envision hybrid approaches that combine crowdsourcing and automated methods to enable fast, comprehensive, and accurate reviews at low cost.

### 2.1 Related work

Over the past decade, crowdsourcing has become an established methodology across a diverse set of domains.^6^ Indeed, researchers have demonstrated the promise of harnessing the “wisdom of the crowd” with respect to everything from conducting user studies^7^ to aiding disaster relief.^8^, ^9^

Perhaps most relevant to the task of citation screening for systematic reviews, crowdsourcing has also been used extensively to collect *relevance judgements* to build and evaluate information retrieval (IR) systems.^10^ In such efforts, workers are asked to determine how relevant retrieved documents are to a given query. In the context of IR system evaluation, crowdsourcing has now been established as a reliable, low cost means of acquiring “gold standard” relevance judgements.^11^ Using crowdsourcing to acquire assessments of the relevance of articles with respect to systematic reviews is thus a natural extension of this prior work. However, the notion of “relevance” is stricter here than in general IR tasks, because of a well‐defined set of inclusion criteria (codified in the specific questions).

A related line of work concerns “citizen science” initiatives.^12^ These involve interested remote, distributed individuals—usually volunteers—to contribute to a problem by completing small tasks. A prominent example of this is the *Galaxy zoo* project,^13^ in which crowdworkers were tasked with classifying galaxies by their morphological features. This project has been immensely successful in turn demonstrating that having laypeople volunteer to perform scientific tasks is an efficient, scalable approach. While we have used paid workers in the present work, we believe that in light of the nature of systematic reviews, recruiting volunteer workers (citizen scientists) may represent a promising future direction.

Indeed, members of the Cochrane collaboration have investigated leveraging volunteers to identify randomized controlled trials.^14^ This project has been remarkable in its success; over 200 000 articles have now been labeled as being randomized controlled trials (or not). Noel‐Stor et al of the Cochrane collaboration have also explored harnessing distributed workers to screen a small set of 250 citations for a diagnostic test accuracy review (Noel‐Stor, 2013). In this case, however, 92% of the workers had some knowledge of the subject matter, which contrasts to the use of laypeople in our project.

The above work has demonstrated that crowdsourcing is a useful approach generally, and for some large‐scale scientific tasks specifically. However, as far as we are aware, ours is the first study to investigate the use of crowdsourcing citation screening for specific systematic reviews to laypersons.

## 3 MATERIALS AND METHODS

### 3.1 Overview

**Figure 1:**
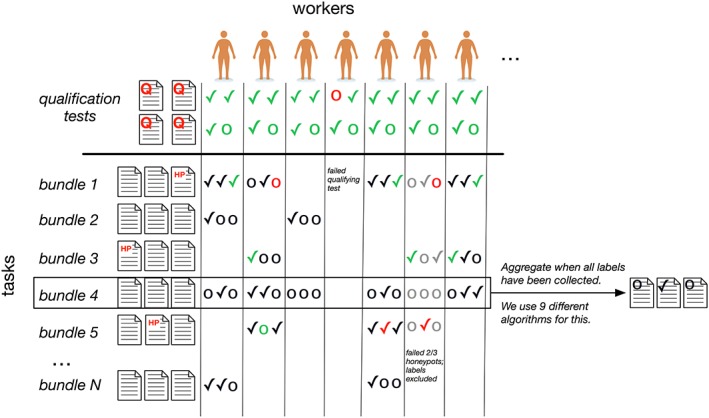
A schematic of the crowdsourcing process used for this work. [Colour figure can be viewed at wileyonlinelibrary.com]

Figure 1 outlines the crowdsourcing experiment. In brief, multiple (usually 5) crowdworkers who passed a qualification test were independently tasked with making decisions about citations with respect to review relevance criteria. These responses were then aggregated to form final relevance decisions. Note that screening in this paper throughout refers to assessing relevance at the *citation* (title, abstract, or keyword) level.

### 3.2 Datasets

Because our goal was to explore the potential of crowdsourcing to facilitate systematic reviews, we used convenience samples of the citations screened for 4 completed or ongoing reviews conducted by our teams at the Center for Clinical Evidence Synthesis at Tufts Medical Center and the Center for Evidence‐based Medicine at Brown University. Specifically, we examined only citations with PubMed records, which were returned from searches pertaining to one of several key questions in a systematic review or to update of the original search; see Table 1. Note that this means the studies to be screened (here, by crowdworkers) already matched a carefully custom‐designed PubMed search query for each review, which codifies the inclusion criteria in a Boolean search string.

**Table 1:** Description of systematic review datasets

Systematic Review Dataset (Reference)	Number of Citations Screened in Full Review, N	Citations Selected in the Experiment a. Description b. Number, n	Honeypots, Number	Screened in at Title/Abstract/Keyword Level (% of n)	Screened in on the basis of Full Text (% of n)
**Proton beam (pilot review):** comparative effectiveness and safety of charged particle radiation therapies for cancer^6^	5208	a. With PubMed records b. n = 4749	0^a^	243 (5.1)	22 (0.5)
**Appendicitis:** diagnostic performance of tests in patients with right lower quadrant pain suspected for acute appendicitis^7^	21 650	a. With PubMed records, identified in updating and published in 2013 b. n = 1664	10	242 (14.5)	61 (3.7)
**DST:** comparative effectiveness and safety of decision aid interventions in people facing screening and treatment decisions for cancer^8^	15 515	a. With PubMed records, identified in updating and published between 2012 and 2014 b. n = 8071	10	183 (2.3)	46 (0.5)
**Omega‐3:** association of omega‐3 fatty acids intake with cardiovascular disease risk factors and outcomes^9^	9676	a. With PubMed records, pertaining to the updated outcomes from a previous report and published between 2002 and 2015 b. n = 5774	10	310 (5.3)	144 (2.5)

a No honeypots were used for quality control in this first review (see text).

One review—on charged‐particle radiation therapy for cancer^15^—was used to test and develop the final experiments by running 7 limited‐scope pilot experiments to refine the format of the questions posed to workers, as well as to address whether to provide training or Supporting Information, whether to use quality control measures, and how much to compensate workers (see Table 2). We refined these items by examining responses to posed questions and analyzing direct feedback provided via emails and indirect feedback through monitoring comments about our projects on online discussion and review boards (eg, mturkforum.com and turkopticon.ucsd.edu). The other 3 systematic reviews pertained to the diagnosis of acute appendicitis^16^; use of decision aids by people facing screening and treatment decisions for early cancer^17^; and associations of omega‐3 fatty‐acid intake and cardiovascular disease risk factors and outcomes.^18^

**Table 2:** Lessons learned from each experimental iteration of citation screening

Experiment	Lessons Learned
**Pilot 1** was our first naive attempt to crowdsource citation screening. For this we created 7 separate questions with possible answers *Yes*, *No*, *Maybe*, and *NA*. The interface showed one citation at a time, there were no qualification tests or honeypots, and all definitions were displayed together at the bottom of the page. Payment was set to $0.50	Quality controls are needed to avoid spamming (ie, low quality and “bare minimum” responses issued to receive payment).
	Answering 7 questions took a lot of time even when the answer to one of the first questions was “No,” which immediately precluded the citation from inclusion, anyway.
	When asking for numerical facts, we can more easily detect errors (and thus spam) by asking for the number rather than a Yes/No answer regarding the number.
	Workers did not understand the point of the NA answer.
	Workers had to scroll down to read definitions often, hurting efficiency and result quality.
	The payment was unnecessarily high.
	Workers lacked a means of providing feedback.
**Pilot 2** was a reiteration of the first kickoff experiment with payment reduced to $0.20. Apart from a slightly decreased completion speed, there was no discernable quality degradation. A few workers did remember being paid $0.50 before and were upset.	Explaining the reasoning behind reducing payment would have been beneficial.
**Exp 1** was the second large iteration, addressing some of the issues discovered after our pilot experiments. The number of questions was reduced to 4 by combining some concepts and the possible answers were changed to *Yes*, *No*, *I cannot tell*, and *The study did not involve humans* (for all but the first question). A qualification test was introduced. Definitions were moved in before the questions they were relevant for. Questions concerning numerical facts were changed to ask for the number rather than ask if the number satisfied our constraints (ie, “How many humans were involved in the study?” rather than “Does the study include at least 10 humans?”). Payment was reduced to $0.10, and a field was added at the end to enable workers to provide feedback if anything was unclear.	Reducing the payment from $0.20 to $0.10 increased the response time, but did not reduce quality.
	While question 2 (Q2), “How many humans were involved in the study?” was easy to answer, very few citations were excluded with this question. Most citations were removed with Q1 followed by Q3, Q4, and finally Q2.
	The interface was hard for workers to read, because it lacked structure.
	While the qualification test reduced the amount of spam, some workers passed the test but still later provided poor‐quality responses.
**Exp 2** addressed the lessons learned from Exp 1. reducing the payment further to $0.05, adding titles before each question, changed the order of questions and introduced honeypots as an extra quality control.	Response was slow, likely partly because of qualification requirements and payment.
	Honeypots drastically reduced spam incidents.
	Workers still reported lack of structure in the interface
**Exp 3** mostly reiterated Exp 2, except that we reduced the payment further to $0.03 and removed the qualification test to improve response time.	The payment of $0.03 was too low, resulting in significant worker backlash both directly and on the Mechanical Turk review site Turkopticon (http://turkopticon.ucsd.edu/).
	Removing the qualification test did not have a significant impact on response time from quality workers, but did induce significant cost for 3 honeypots per spammer, which was needed to determine if a worker should be blocked.
For **Exp 4** we completely reconfigured the interface to one that focuses especially on optimizing efficiency and minimizing cost. Instead of one citation per HIT, each HIT had 3 citations, thus reducing time lost in the Mechanical Turk submission process. The qualification test was reintroduced alongside the honeypots. Automated logic was added to the interface such that answering *No* to any question automatically redirected the worker to the next citation. Likewise answering *Yes* or *I cannot tell* brought the worker to the next question, showing only definitions relevant to each question at a time. Payment was set to $0.10 given the optimizations and bundling of 3 citations.	Despite the optimizations improved worker income per hour, the payment was still too low. As a result, similar worker backlash occurred and response time was poor.
	Some workers were reading the citations in full before giving responses, thus heavily impacting response time. Some even tried to understand each medical concept before answering, to avoid making mistakes.
	Instructions for how to complete the citation screening were not clear enough and left too many details up to the worker (eg, how to quickly determine a firm *No* to a question).
	Instructions gave no insight into the mechanics of citation screening, thus making workers fear many citations were actually relevant, despite the worker thinking the answer to a question was *No*.
**Exp 5** addressed the lessons learned in Exp 4. Specifically, we expanding on task background descriptions (eg, “Only 5%‐10% of abstracts are likely includes”), displayed tips for working efficiently (eg, “Only read enough to answer each question in order,” “Don't try to understand the concepts, try instead to look for textual patterns”). We also increased the payment to $0.15 and explained that including a citation incorrectly was significantly better than excluding a citation incorrectly. Finally, to ensure transparency, we added a Brown University logo as well as direct contact and affiliation information.	The price point of $0.15 improved response time again; however, some workers were still not performing efficiently enough, resulting in low hourly pays for those workers.
	Some workers still did not understand what we were studying and questioned the purpose of the work.
**Proton beam—full‐scale experiment.** At this point the changes needed from Exp 5 were small enough to merit issuing the full‐scale experiment with 4749 citations. For the full‐scale experiment we added a more thorough explanation of citation screening in the context of evidence‐based medicine, the purpose of our research and how to do the work efficiently with “dos and don'ts”. We also removed the honeypots to cut costs, because the last few initial experiments showed no situations in which workers were being blocked by these hidden tests. The logic was that because we had run several iterations all the spammers had already been blocked and most of our workers were recurring, with the remaining being deterred sufficiently by the qualification test and our achieved reputation on Turkopticon.	Removing the honeypots was a mistake. Knowing no hidden tests were present, a few workers began providing erroneous responses and began answering *No* to Q1 on all citations because that made finishing HITs as quick as possible. These spamming workers were subsequently blocked, and we reintroduced honeypots in subsequent full‐scale experiments.
**Suggested future improvements**	The explanation in the beginning of each HIT is useful when the worker is not aware of the purpose. After having read it a few times however, it just clutters and creates the need to scroll for each hit. Removing it will improve response time further.
	Some workers have expressed a wish to be retrained when sufficient time has passed between experiments. One possible option here would be to introduce a nonpaid training step in the start of each experiment cycle. This would give experienced workers the opportunity to have their skills refreshed before working on actual citations.
	Some workers have misunderstood our auto‐approval of their HITs as a seal of approval of the correctness of their answers. A better description of the purpose of honeypots and the approval process could possibly solve this issue.
	Some workers have expressed disapproval with the conditional questions in our DST experiment. Specifically, they found the bundling of several questions and conditionals into one question confusing, eg, “IF this study is about patients, is it a randomized controlled trial (RCT) with at least 10 participants in each group, OR, IF the study is about providers, is it a study with some form of a comparison aspect (eg, RCT, but also nonrandomized groups, before/after comparisons, etc)?”
	A conditional interface, dynamically showing the relevant questions depending on worker answers, may be a solution.
	To further avoid low‐quality workers, one could automatically flag a worker as questionable if his/her completion time per HIT is unrealistically low. Such a flagging could be used to temporarily block the worker until answers have been evaluated manually.

The following subsections describe lessons learned from each experimental iteration of citation screening the Proton beam dataset. The final interface and processing and quality controls were developed over several months during the summer of 2014. We note that this preliminary work was necessary because no prior work on crowdsourcing citation screening existed. Once we settled on our setup and interface, comparatively little effort was needed to begin acquiring crowd labels for citations from new datasets.

We selected these 4 topics because they span different questions (treatment versus diagnosis; cancer versus infection versus cardiovascular disease; radiation therapy versus quality improvement intervention versus nutrient intake) and thus may pose different degrees of difficulty to nonexpert workers.

### 3.3 Crowdsourcing setup and evaluation

We conducted experiments using the Amazon Mechanical Turk platform (http://www.mturk.com/). We used Mechanical Turk, as opposed to alternative crowdsourcing platforms, because it is the most widely used; however, we have designed our approach in such a way that we believe it will generalize to other platforms. Mechanical Turk provides easy access to a large pool of available workers, has built‐in payment and worker systems that make managing and compensating workers easy, and features an extensive application programming interface, which enabled us to add functionality by incorporating quality controls, qualification tests, provide additional information on demand and more.

Work on Mechanical Turk is organized into sets of human intelligence tasks (HITs). Crowdworkers can search for and accept work on sets of HITs, some of which may require passing a qualifying test. Once they are deemed qualified to perform the task, they are presented with a set of HITs to complete sequentially. In our case, each HIT comprised a bundle of 3 citations to be screened. When workers submit each HIT, their answers are sent to the work provider (the requester) who may either accept the answers if they meet the HIT instructions or reject them if they do not. Accepting the answers results in a payment to the worker for that HIT. Mechanical Turk provides automatic acceptance mechanisms and other quality control measures.

In this work we used Amazon Mechanical Turk workers to provide services. This research was determined by the Brown Human Research Protection Program to not meet the definition of human subjects' research as defined in Title 45 CFT Part 46.102(f); thus, no IRB approval was deemed necessary. Crowdworkers were informed that the work they were doing was part of a study. To guide the development of fair work requests on Mechanical Turk, we relied on the *Guidelines for Academic Requesters* 19 document developed by crowdsourcing researchers. At the time of this writing (October 2016), these guidelines had been “signed” by nearly 70 academic researchers and over 180 experienced Mechanical Turkers. In particular, following these guidelines, we clearly identified ourselves and provided a direct line of contact; provided fair payment (as defined by the guideline); provided reasonable time estimates/limits; and avoided unfair rejections and approved work as promptly as possible.

### 3.4 Citation screening HIT structure

In recent years the Mechanical Turk worker population has shifted from a primarily US‐based moderate‐income population toward an increasingly international group, including young, well‐trained people from developing economies.^20^ We conjectured that most Mechanical Turk workers are unlikely to have substantial medical expertise. We therefore had to take some care in designing HITs for this specialized task. It was unlikely that simply providing inclusion criteria and asking for an overall decision on each citation would work. Instead, we decomposed the eligibility criteria for each review into sets of simple successive pattern‐matching and information‐extraction questions regarding study eligibility subcriteria. Questions were devised so that they required minimal understanding of the contextual or methodological (eg, study design) issues. These simplifications effectively corresponded to a (slight) broadening of the citation screening criteria as compared to screening criteria used by trained experts.

For example, for each systematic review, we first asked workers to infer whether the abstracts implied that the corresponding article described a primary study of humans or not. The possible responses were *Yes*, *No*, and *I Cannot Tell*. *Yes* and *I Cannot Tell* indicated possible inclusion, while *No* indicated definite exclusion, regardless of answers to subsequent questions. If a worker answered *No* for any question in a particular citation, she was not asked any additional questions about that citation. If she answered *Yes* or *I Cannot Tell*, the next question was presented until a question was answered with a *No* or all of the questions had been answered. We ordered these subquestions in (estimated) descending order of prevalence, such that common reasons for exclusion were encountered first, thereby economizing worker effort by minimizing the number of questions considered per worker. Because worker screening decisions are inexpensive and perhaps noisy (prone to error), we collected 5 independent label sets for each citation.

To support workers in making their decisions, we provided definitions of technical terms and created illustrative positive and negative examples of subcriteria (see Appendix B). Using these materials we attempted to explain the necessary concepts with as little medical jargon as possible, ideally by identifying terms that workers could look for in the abstracts (without necessarily understanding their full meanings).

We “bundled” 3 citations into a single HIT to minimize the time lost in switching between HITs, and to increase the reported compensation per HIT. We paid $0.15 to $0.21 for each bundle of 3 citations. (We later report estimated effective hourly wages in Table 6).

### 3.5 Quality controls

To encourage quality responses and limit unconscientious workers, we relied on 2 standard quality control mechanisms: *qualification tests*, an internal Mechanical Turk mechanism, and hidden gold‐standard control tests, commonly referred to as *honeypots.* 21

*Qualification tests* are natively supported by Mechanical Turk and involve tasking workers with a set of unpaid representative tasks that evaluate their ability to answer the HITs correctly. In our experiments, we provided workers with 4 citations to screen, ranging in difficulty from clear‐cut cases to challenging, borderline cases. Workers were expected to answer all of the questions correctly, although they were allowed multiple attempts with occasional manually provided feedback. Upon completion of the qualification test, workers were allowed to work on all future citation screening HITs, including HITs for other systematic reviews.

We also injected hidden control tests, commonly referred to as *honeypots*, among regular citations in HITs early in the screening process, to identify and eliminate unconscientious workers. (We provide technical details on the acceptance, rejection, and injection of honeypots in Appendix D.) The honeypots were citations for which we had domain experts provide answers to each question. These were used to automatically evaluate worker performance against the supplied answers. If a worker answered all honeypot questions correctly, screening continued uninterrupted (and the worker was never made aware of the honeypot). If, however, they answered one or more questions incorrectly, they were informed of their error, what the right answer was (and why that was the right answer), and they were warned that additional failed honeypots may result in exclusion from participation in our HITs. Workers passing at least 2 of 3 honeypot tests were allowed to continue; others were disqualified.

While this approach to quality control may seem overly stringent (possibly annoying Turkers), workers generally spoke positively about the training and automated feedback benefits of the honeypot testing. For selected comments from workers, see Appendix C.

We did not use honeypots in the charged particle radiation therapy dataset, as our interpretation from the pilot experiments suggested that they were superfluous (see Table 2). Upon analysis of the results from this dataset, however, we realized that we had received a significant amount of careless, wrong responses (ie, *No* to the first question regardless of content) from a small subset of workers. Therefore, we decided to include honeypots for the remaining 3 full‐scale experiments. Potentially malicious workers were subsequently blocked and their responses excluded.

We note that here we have aimed to develop and implement a practical crowdsourcing strategy to evaluate the potential of this approach for citation screening. We did not aim to exhaustively explore these design options. We did not, for example, check worker IP addresses or limit responses from a given IP. And we did not make use of quality control mechanisms specific to the Mechanical Turk platform (for example, we did not hire only workers with “Masters” qualifications) because we wanted to evaluate a general strategy that could be used on most crowdsourcing platforms.

### 3.6 Example: systematic review on the diagnosis of acute appendicitis

**Figure A1:**
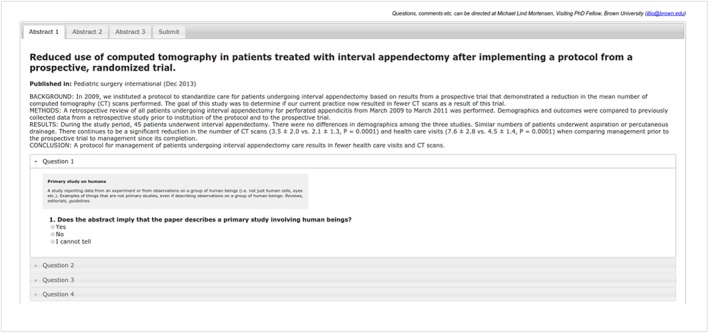
Appendicitis review, citation screening HIT interface. [Colour figure can be viewed at wileyonlinelibrary.com]

We performed experiments for the 4 datasets summarized in Table 1. The following subsection presents an example of citation screening for the Appendicitis dataset. For each HIT, workers were presented with a bundle of 3 citations, accessible through the tabs at the top of the interface window in Figure A1.

The first question we asked for each citation was (*1*) *Does the abstract imply that the paper describes a primary study involving human beings?* We provided definitions of a primary study and exceptions for studies on parts of humans (eg, previously removed appendixes). If the worker answered *Yes* or *I cannot tell*, the next question was displayed. If the worker answered *No*, the interface immediately switched to the next citation. The remaining questions for each citation were, in order: (*2*) *How many humans were involved in the study?* (*3*) *Does the abstract imply the patients had right lower quadrant (or abdominal) pain of less than seven days duration, had suspected appendicitis, or underwent treatment for appendicitis?* and (*4*) *Does the abstract imply that the paper studies testing/diagnosis methods rather than treatments?* Again, definitions and exceptions were provided for each concept.

**Figure A2:**
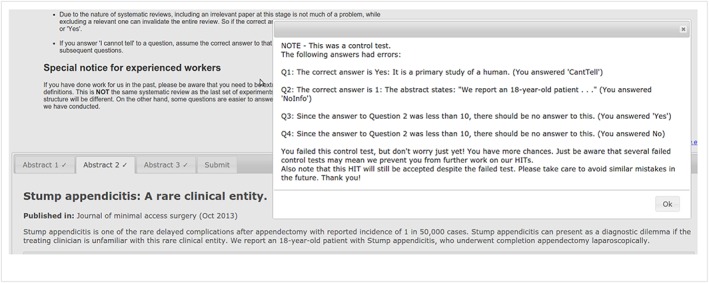
Appendicitis review, honeypot failure with feedback for workers. [Colour figure can be viewed at wileyonlinelibrary.com]

Upon completion of all 3 bundled citations in an HIT, workers were shown a submission page. This page allowed workers to provide feedback if anything was unclear. If none of the citations were an injected honeypot (or if the worker passed the honeypot), then the answers were sent to us. If, however, the worker failed a honeypot, the answers were sent to us, and a message regarding the mistakes was shown to the worker along with a warning to avoid similar errors in the future or risk being blocked from working on the remaining HITs. We show an example of such a honeypot in Figure A2, where a worker answered questions incorrectly.

After we had collected 5 crowd responses for each citation, we examined 9 aggregation strategies for deriving final answers regarding citation relevance. Results for blocked workers were removed before their application (Table 3). The first 8 aggregation strategies consider each question separately:

1. **Majority**—For each question, choose the answer most workers assigned.
2. **1p**—For each question, assume *Yes* if at least 1 worker says *Yes* or *I cannot Tell*.
3. **2p**—For each question, assume *Yes* if at least 2 workers say *Yes* or *I cannot Tell*.
4. **3p**—For each question, assume *Yes* if at least 3 workers say *Yes* or *I cannot Tell*.
5. **4p**—For each question, assume *Yes* if at least 4 workers say *Yes* or *I cannot Tell*.
6. **5p**—For each question, assume *Yes* if all 5 workers answered *Yes* or *I cannot Tell.*
7. **Champion**—Works the same as the majority rule, except that the majority decision is based solely on those who actually answered each question. For example, workers answering *No* to question 1 are not assumed to have also answered *No* to subsequent questions.
8. **Champion (DR)**, that is Champion rule with decreased requirements—Works the same as the Champion rule, except that for each question we reduce the requirement for inclusion (ie, assuming 5 workers, we required 3 *Yes* or *I cannot tell* answers for question 1 (majority), 2 answers for question 2, and 1 answer for questions 3 and 4 (again, ignoring workers who did not evaluate later questions because of an early *No* answer).

**Table 3:** Example clarifying the 9 the aggregation strategies

	Crowdworkers					Aggregation Strategy								
	W1	W2	W3	W4	W5	Majority	p1	p2	p3	p4	p5	Champion	Champion (DR)	Majority Question
Q1	Yes	Yes	Yes	Cannot tell	No	Yes	Yes	Yes	Yes	Yes	No	Yes	Yes	12 Yes or Cannot Tell/20 maximum answers^b^
Q2	Yes	Yes	Yes	No	—a	Yes	Yes	Yes	Yes	No	No	Yes^b^	Yes^b^	
Q3	Yes	Yes	Yes	—a	—a	Yes	Yes	Yes	Yes	No	No	Yes^b^	Yes^b^	
Q4	Yes	Yes	No	—a	—a	No	Yes	Yes	No	No	No	Yes^b^	Yes^b^	
Citation screened in?	Yes	Yes	No	No	No	No	Yes	Yes	No	No	No	Yes	Yes	Yes

Q1‐Q4 means question1 through 4. W1‐W5, means crowdworkers 1 through 5. In this example, using the p1, p2, Champion, Champion (DR), or Majority Question aggregation algorithms would have resulted in the citation being screened in. Using p3, p4, or p5 would have led to exclusion.

a Question not posed because a previous answer was *No*.

b Imputing *No* for the questions that have not been posed because of a previous *No* answer.

The ninth strategy ignores stratification of responses by question and examines responses of all raters to all questions together:

1. **Majority question**—Consider all answers together regardless of the questions and include those citations where most answers are *Yes* or *I cannot Tell.* Workers who answered “No” to an early question are assumed to have answered “No” to any subsequent questions (which, by design, were not posed to them).

### 3.7 Measuring performance

In using crowdsourcing to facilitate citation screening, there are 2 objectives. The first is to maximize the proportion of identified citations among those that were included in the systematic review by trained experts (our reference standard). We quantify this objective as the sensitivity of the crowdsourcing strategies in with respect to identifying relevant citations (as decided upon full‐text screening); we refer to this as *yield*. The second objective is to minimize the proportion of irrelevant citations that an expert would have to review in full text. This is the complement of the specificity with respect to title/abstract/keyword screening. We call this quantity *gain*.

We also compared the cost of obtaining crowdsourced decisions using each of the 9 aggregation strategies with an approximation to the actual cost incurred using trained experts to screen the same number of citations at the citation level. We calculated the cost of using trained experts, assuming that it takes them 30 seconds on average to screen a citation (an estimate on the basis of observations from our own experience), and using approximate hourly costs commensurate with the salaries of the systematic reviewers who performed the majority of the citation screening in each project. Costs have not been translated to 2015 US dollars and are only approximately comparable. We report figures both including and excluding fringe costs.

## 4 RESULTS

**Figure 2:**
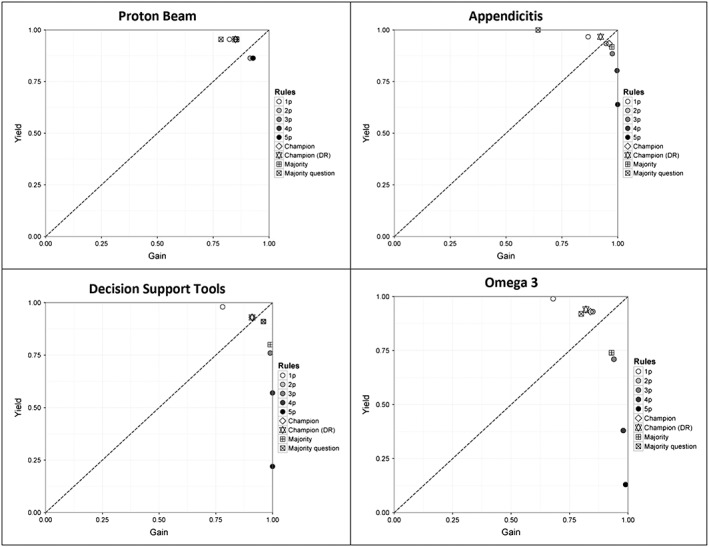
Results on each dataset using the 9 aggregation strategies

**Figure A3:**
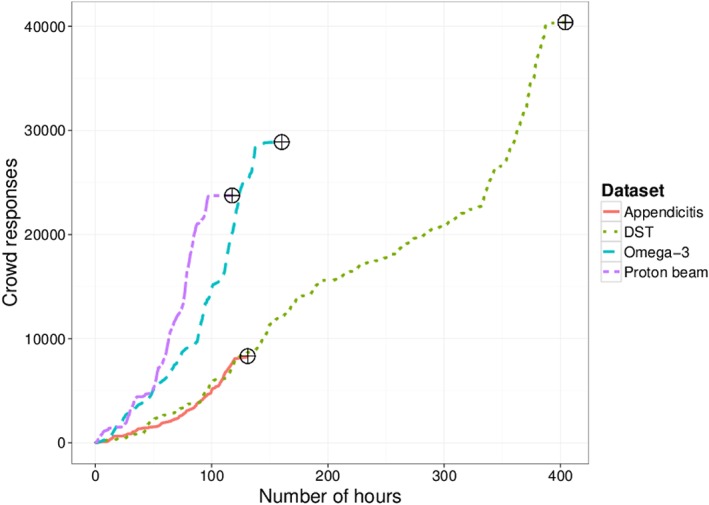
Time elapsed (hours) vs number of crowd screening decisions received. [Colour figure can be viewed at wileyonlinelibrary.com]

We achieved high yield for relevant articles using crowdsourced decisions (compared to manual screening), although at some cost in gain. Figure 2 shows these results graphically for the 9 aggregation strategies. The most conservative approach (*1p rule*, in which we consider a citation relevant if any of the 5 workers screened it in) achieved a yield range of 95% to 99% with corresponding gain ranging from 68% to 82%. Less stringent criteria for exclusion increased gain but decreased yield. For example, taking a simple majority vote for citation relevance across workers (majority rule) lead to a yield of 74% to 95% and a gain of 86% to 99% (Table 4 and Figure A3).

**Table 4:** Experimental results for the 9 aggregation strategies across the 4 datasets

Dataset	Performance of Aggregation Strategies for Crowdworker Answers (Yield; Gain)								
	Majority	1p	2p	3p	4p	5p	Champion	Champion (DR)	Majority Question
Proton beam	0.95; 0.86	0.95; 0.82	0.86; 0.92	0.86; 0.93^a^	0.86; 0.93	0.86; 0.93	0.95; 0.85	0.95; 0.85	0.95; 0.78
Appendicitis	0.92; 0.97	0.97; 0.87	0.93; 0.95	0.89; 0.98	0.80; 0.99	0.64; 1.00	0.93; 0.96	0.97; 0.92	1.00; 0.64
DST	0.80; 0.99	0.98; 0.78	0.91; 0.96	0.76; 0.99	0.57; 1.00	0.22; 1.00	0.93; 0.91	0.93; 0.91	0.93; 0.91
Omega3	0.74; 0.93	0.99; 0.68	0.93; 0.85	0.71; 0.94	0.38; 0.98	0.13; 0.99	0.93; 0.84	0.94; 0.82	0.92; 0.80

a Lack of improvement after 3p due to a small number of unconscientious workers in the pool. In the proton beam dataset we did not use honeypots as a quality control mechanism (see text).

We report Fleiss Kappa scores for each review (calculated independently for each question) in Table 5.^22^ One can observe quite a bit of variance; agreement ranges from poor to moderate. As worker‐agreement deteriorates, tougher exclusion policies (eg, honeypots) and/or more conservative exclusion strategies are needed to ensure high yield. These results suggest that further research into minimizing disagreement is warranted. Note however that poor agreement on individual questions does not imply poor overall crowd performance; indeed, using recall‐centric aggregation strategies, we will later demonstrate that despite this ostensibly low per‐question agreement we are able to achieve strong performance with respect to abstract‐level inclusion/exclusion decisions. Consider the scenario of an irrelevant citation for which multiple individual questions could yield a *No*, but where such determination may be more unclear than a *Yes* for a relevant citation. Some workers may correctly determine *No* to be the right answer for the first question, while others say *I cannot tell* or play it safe with a *Yes*; only later stating *No* to the subsequent questions the first worker did not address. This creates clear disagreement on the level of individual questions, as exposed in Fleiss Kappa scores, but this disagreement is of little consequence if the end result is the same. Both workers will eventually determine the citation irrelevant. Even in the extreme scenario where all citations are irrelevant and correctly determined as such, the Kappa Fleiss results could still show significant disagreement on an individual question level.

**Table 5:** Fleiss kappa (a measure of agreement) calculated for each question

Dataset	Q1 Kappa	Q2 Kappa	Q3 Kappa	Q4 Kappa	Average Kappa
Appendicitis	0.252	0.500	0.387	0.196	0.333
DST	0.056	0.057	0.018	−0.030	0.026
Omega3	0.245	0.203	0.116	NA	0.188
ProtonBeam	0.175	0.128	0.063	0.071	0.109

Average agreement ranges (across reviews) from slight to fair, motivating the use of aggregation strategies.

As can be seen in Tables 6 and 7, crowdsourced screening decisions were relatively inexpensive compared to the usual screening process. Furthermore, as illustrated by Figure A3, leveraging the crowd can enable relatively rapid screening decisions. For example, within 100 hours approximately 15 000 screening decisions were made by crowdworkers for the Omega‐3 review. We note that the DST and Omega‐3 HITs were made available on Mechanical Turk semiconcurrently, which may explain the slower pace of screening for the former (presumably because Omega‐3 paid a bit more). Once the Omega‐3 project was completed, we saw a sharp rise in crowd responses per hour for DST.

**Table 6:** Costs and duration of each crowdsourcing experiment

Dataset	Worker Salary (with Amazon fee^a^)	Approximate Cost of Experts' Screening (with Fringe^b^)	Experiment Running Time (after Task Setup)
Proton beam	$1187.25 ($1305.98)	$6859.67 ($8917.57)	4 d, 21 h, and 36 min
Appendicitis	$416.00 ($457.60)	$3034.23 ($3944.50)	5 d, 10 h, and 58 min
DST	$2017.75 ($2219.53)	$6173.75 ($8025.88)	16 d, 20 h, and 11 min
Omega3^c^	$2020.90 ($2222.99)	$8776.79 ($11 409.83)	6 d, 16 h, and 17 min

a At the time we ran our experiments, Amazon Mechanical Turk charged a 10% commission fee on each HIT, with a minimum payment of $0.005 per HIT; this has since been increased to 20% (https://requestersandbox.mturk.com/pricing).

b Fringe benefit costs are estimated here to be 30% of salary, reflecting (roughly) the true costs at the institutes at which this work was performed (Tufts and Brown).

c Because of the higher complexity of questions for this review, worker compensation was increased from $0.15 to $0.21 per HIT

**Table 7:** Estimated hourly pay rates for workers, using different thresholds to infer when workers were not actively working. See text for discussion

Dataset	30m	15m	10m	5m
Appendicitis	$3.73	$3.94	$4.15	$4.41
DST	$3.60	$4.06	$4.31	$4.97
Omega3	$6.25	$6.45	$6.75	$7.44
ProtonBeam	$5.89	$6.29	$6.40	$7.08
Average overall	$4.87	$5.18	$5.40	$5.97

The plateau effect seen in Figure A3, when screening approaches completion, is due to workers evaluation of their potential maximum payment. If there are only a few HITs available, many workers invest their time elsewhere.

When workers submitted answers to HITs, we included a timer to keep track of time used per HIT. There are 2 main limitations of that approach: (1) Time used is for the entire HIT (which comprises 3 abstracts), rather than separated per abstract. (2) We cannot determine if a worker is concentrating, multitasking, or taking a break between the abstracts that comprise a single HIT. To approximate an hourly wage, we must therefore use heuristics to infer actual work time. To this end we removed values exceeding the cutoff point before taking an average. In Table 4 we report the estimated hourly wage paid to workers, along with the cutoff points used. Note that the average hourly wage for a cutoff point of 5 minutes is roughly equivalent to the recommended minimum from *guidelines for academic requesters.* Given that domains experts spend approximately 30 seconds to screen 1 abstract (on average), a cutoff point of 5 minutes seems reasonably conservative.

## 5 DISCUSSION

Crowdsourcing may represent a useful approach to reducing the workload involved in conducting systematic and scoping reviews. By collecting redundant decisions for each citation and aggregating these, we were able to derive relatively high‐quality screening decisions at low cost. As the number of published articles continues to explode, evidence syntheses are going to become increasingly important, but also increasingly expensive and time‐consuming. Text mining and crowdsourcing methods that reduce the financial and time burden of the more mundane, but still critical, aspects of systematic review production will be increasingly valuable as they are developed, improved, and eventually adopted in practice.

This study presents the first empirical evaluation of crowdsourcing citation screening for eligibility in a systematic review. It includes reviews for which we have domain expertise, so we are able to provide detailed explanations and feedback to workers as questions arose. We also ran preliminary tests to refine our instruction sets, which required time, knowledge, and expertise.

### 5.1 Study limitations

Our study also has several limitations. Although we selected the 4 systematic review datasets to be diverse, the number is still small to generalize results. We used a systematic convenience sample of the citations screened in each systematic review. For each project, we examined only citations with PubMed records, the subset of citations identified during the updating phase of systematic reviews, or the citations pertaining to one of several key questions. Our intent was to limit the number of citations that had to be screened and thus to cap the amount of money spent for crowdsourcing in this first experimental foray. However, we cannot identify a plausible mechanism by which these choices systematically bias our results. The 4 topics were examined sequentially, and know‐how from the first (proton beam) was used in the setup of the other three, corresponding to differences in the execution of the 3 experiments. Nevertheless, we refrain from making strong claims about how these results generalize.

Crowdsourcing using the 9 aggregation strategies failed to identify all the papers that were eligible upon full‐text screening (yield was less than 100%), which is concerning given the emphasis on comprehensiveness in systematic reviews (although we note that human screeners are not infallible). In looking at the citations that were consistently missed by all screeners, we found no obvious explanation as to why the citations were incorrectly labeled as irrelevant. It may be that the questions, which had been simplified to make them more accessible to lay evaluators, were not clear enough to distinguish borderline includes, or that instructions to include when in doubt were not stated clearly enough. Further redundancy (ie, more workers per citation) could potentially have caught these false excludes, as could the use of human‐machine hybrid approaches.

It is conceivable that combining our approach with text classification approaches for semiautomating citation screening^3^ could yield greater sensitivity, because a computer model could determine strong inconsistencies between worker answers and model expectations, indicating borderline citations in need of expert annotation. However, several strategies showed high sensitivity (above 90%) across all 4 topics, and this performance may be good enough for scoping reviews, in which it is expected to identify most but not necessarily all relevant papers.

## 6 CONCLUSIONS AND FUTURE RESEARCH

Given the relatively high accuracy and comparatively low cost of crowdsourced screening over these 4 systematic review projects, further research in this direction is warranted. For example, it will be important to replicate our results here using other datasets. One open question is whether similar results can be achieved in other research areas.

Beyond replication and assessment of the generalizability of the approach, inserting additional quality control mechanisms into the process to identify problematic workers may substantially improve results. One may, eg, attempt to recognize and exclude “streakers,”^23^ ie, individuals who submit many labels in quick succession. With no prior screening process, such individuals may negatively affect quality and price.

There is also a natural question regarding the trade‐off between investing the time to design quality assurance tests upfront versus using models to recognize and exclude unreliable workers posthoc. Here we have favored the former approach, but we believe there is merit to the latter strategy. In general, investigation and evaluation of more sophisticated methods for statistical aggregation of individual worker decisions (which account for estimated worker reliability) across the inclusion criteria questions will provide important additional data and potentially mitigate quality control concerns. This is therefore a promising direction to explore in our view.

Beyond better worker quality models for label aggregation, we believe a promising research direction concerns “hybrid” human‐machine screening processes. In particular we foresee domain experts, crowdworkers, and machine‐learning algorithms working in concert to screen and synthesize literature. Our initial work^24^ has highlighted the potential of this approach, but many open questions remain.

Finally, we note that, as with all approaches that rely on crowdsourced work, there are clear ethical concerns here. In particular, there is concern regarding fair worker compensation. Naturally, it is our view that systematic review teams outsourcing any screening effort to crowdworkers should pay fair wages to workers. In the present work we have done just this and still managed to keep costs down. In particular, using conservative estimates (reported in Table 7) we paid the equivalent of $5 to $7 per hour; this is only slightly below US federal minimum wage, and substantially higher than minimum hourly wages paid in many developing countries, *
https://en.wikipedia.org/wiki/List_of_minimum_wages_by_country
 where Mechanical Turk workers may live (Turk is an international platform, and we did not discriminate on the basis of nationality). In future work, and if the strategy were to be adopted in practice, it may be advisable to restrict the task to workers residing in countries in which average wages are sufficiently low to render the rate paid here competitive. In any case, as evidenced by the feedback we received (presented in Appendix D), workers found the experience of completing our tasks to be positive. Therefore, while we acknowledge that there is risk for exploitation of workers, we also believe that this approach can beneficial both to systematic review teams and to crowdworkers.

## APPENDIX A—FIGURES

### A—FIGURES.1

The first question we asked for each citation was: (1) *Does the abstract imply that the paper describes a primary study involving human beings?* We provided definitions of a primary study and exceptions for studies on parts of humans (eg, previously removed appendixes). If the worker answered *Yes* or *I cannot tell*, the next question was displayed. If the worker answered *No*, the interface immediately switched to the next citation. The remaining questions for each citation were, in order: (2) *How many humans were involved in the study?* (3) *Does the abstract imply the patients had right lower quadrant (or abdominal) pain of less than 7 days duration, had suspected appendicitis, or underwent treatment for appendicitis?* and (4) *Does the abstract imply that the paper studies testing/diagnosis methods rather than treatments?* Again, definitions and exceptions were provided for each concept.

## APPENDIX B—CITATION SCREENING CROWD QUESTIONS

### B—CITATION SCREENING CROWD QUESTIONS.1

#### Proton beam

**Table:**

**Primary study on humans** A study reporting data from an experiment or from observations on a group of human beings (i.e. not just human cells, eyes etc.). Note that a review is not a primary study, even if describing observations on a group of human beings.

1. Does the abstract imply that the paper describes a primary study involving human beings?
  **Table:**
  **Conditions considered Cancer** The operational definition of cancer includes histologically malignant tumors. Examples are: 1. Liver, lung, prostate, breast etc cancer. 2. Ocular (eye) melanoma == uvular melanoma 3. Head and neck cancer **Conditions NOT considered Cancer:** 1. arteriovenous malformations 2. benign meningiomas 3. benign schwannomas 4. craniopharyngioma 5. age‐related macular degeneration	**Treatments considered External radiotherapy using charged particles** A machine shines a beam from outside the patient's body. The beam consists of charged particles, examples of which include: 1. Hydrogen ion == hydrogen nuclei == protons 2. Helium ions == alpha particles 3. Heavier ions such as carbon‐, neon‐, silicon‐, ferrous‐ 4. Hadrons, is another synonym **Treatments NOT considered External radiotherapy using charged particles** 1. Non‐external radiotherapy such as brachytherapy, i.e., implantation of radioactive seeds in or next to the tumor 2. External radiotherapy with non‐charged particles, e.g., a. Neutrons (particles, but not charged) b. Photons, e.g., gamma rays or X‐rays c. Electrons d. *π*‐mesons
2. Were people treated for cancer with external radiotherapy using charged particles?
  **Table:**
  **Treatment harm** Harms that are related to the treatment. Examples include nausea, vomiting, hair loss, colitis, dry mouth, blood abnormalities, secondary cancers, growing redundant body parts (e.g., a second head)	**Clinical outcomes** Examples include death, survival, recurrence, local tumor control, change in symptoms.
3. Does the abstract imply that the paper reports clinical outcomes or treatment harms?
4. How many humans were involved in the study?

#### Appendicitis

**Table:**

**Primary study on humans** A study reporting data from an experiment or from observations on a group of human beings (i.e. not just human cells, eyes etc.). Examples of things that are not primary studies, even if describing observations on a group of human beings: Reviews, editorials, guidelines.

1. Does the abstract imply that the paper describes a primary study involving human beings?
2. How many humans were involved in the study?
  **Table:**
  **Appendicitis** Appendicitis is an inflammation of the appendix, a finger‐shaped pouch that projects from your colon on the lower right side of your abdomen. The appendix doesn't seem to have a specific purpose. Appendicitis causes pain in your lower right abdomen. However, in most people, pain begins around the navel and then moves. As inflammation worsens, appendicitis pain typically increases and eventually becomes severe. Although anyone can develop appendicitis, most often it occurs in people between the ages of 10 and 30. Standard treatment is surgical removal of the appendix through an appendectomy. (Source: http://www.mayoclinic.org/diseases‐conditions/appendicitis/basics/definition/con‐20023582 *)* **Right lower quadrant (or abdominal) pain** The human abdomen is divided into quadrants to localise pain and tenderness, scars, lumps and other items of interest. The quadrants are referred to as the left lower quadrant (LLQ), left upper quadrant (LUQ), right upper quadrant (RUQ) and right lower quadrant (RLQ). Example mentions of RLQ (or abdominal) pain include: *right lower quadrant pain, right upper quadrant pain, abdominal pain, pain in the abdomen, stomach pain, tummy pain and pain in the midriff*
3. Does the abstract imply the patients had right lower quadrant (or abdominal) pain of less than 7 days duration, had suspected appendicitis or underwent treatment for appendicitis?
  **Table:**
  **Testing/Diagnosis methods** Any method or procedure meant to enable a diagnosis of a new case of appendicits. Exclude recurrent/repeat appendicitis, e.g., in people who have already been treated for appendicitis with conservative (non‐surgical) interventions. **NOTE:** For the purposes of this review, we consider **Laparoscopy** and **Laparotomy** as diagnostic procedures. A laparoscopy is a type of surgical procedure in which a small viewing device (laparoscope) is inserted in the abdomen through small incisions. This allows the doctor to examine the abdominal and pelvic organs on a video monitor connected to the tube. Laparotomy involves a larger incision so that the surgeon can examine the appendix without a camera and viewing tube. (Source: http://medical‐dictionary.thefreedictionary.com/laparoscopy *)*
4. Does the abstract imply that the paper studies testing/diagnosis methods rather than treatments?

#### DST

**Table:**

**Patient types** We are interested in the following patient types 1. Patients with early stage cancer considering treatment 2. Healthy patients considering screening 3. Very high risk patients considering treatment for genetic syndromes like: BRCA genes (breast cancer), Lynch syndrome, MMR genes (colon cancer), FAPC (colon cancer) 4. Patients with family histories suggesting any of the above mentioned genetic syndromes **EXCLUDE** abstracts which study: *Hypothetical scenarios/patients, advanced cancer stages (only if explicitly mentioned as advanced. If in doubt, include), end of life decisions, palliative care (relief treatment)* **Primary study on humans** A study reporting data from an experiment or from observations on a group of human beings (i.e. not just human cells, eyes etc.). Examples of things that are not primary studies, even if describing observations on a group of human beings: *Reviews, editorials, guidelines.*

1. Does the abstract imply the article is a primary study on patients of any of the above mentioned types, **OR**, alternatively, a primary study of providers of care for these patients?
  **Table:**
  **RCT ‐ Randomized controlled trial** A study in which a number of similar people are randomly assigned to 2 (or more) groups to test a specific drug or treatment. One group (the experimental group) receives the treatment being tested, the other (the comparison or control group) receives an alternative treatment, a dummy treatment (placebo) or no treatment at all. The groups are followed up to see how effective the experimental treatment was. Outcomes are measured at specific times and any difference in response between the groups is assessed statistically. This method is also used to reduce bias. (Source: https://www.nice.org.uk/glossary?letter=r)
2. **IF** this study is about patients, is it a randomized controlled trial (RCT) with at least 10 participants in each group, **OR**, **IF** the study is about providers, is it a study with some form of a comparison aspect (e.g. RCT, but also non‐randomized groups, before/after comparisons etc)?
  **Table:**
  **Special case exclusion** Please exclude studies about methods to increase screening participation rates in a population. **Shared decision‐making** Shared decision‐making is a model of patient‐centered care that enables and encourages people to play a role in the management of their own health. It operates under the premise that, armed with good information, consumers can and will participate in the medical decision‐making process by asking informed questions and expressing personal values and opinions about their conditions and treatment options. (Source: https://cahps.ahrq.gov/Quality‐Improvement/Improvement‐Guide/Browse‐Interventions/Communication/Shared‐Decision‐Making/index.html)
3. Are the participants facing a decision about whether to get screening or treatment, **OR** is the study about a provider‐targeted intervention to increase shared decision‐making?
  **Table:**
  **Decision Support Tool (DST) / Decision Aid** Decision Support Tools (DST) provide information on the options and the expected relevant outcomes and implicit methods to clarify values. It may also include information on the health condition, personalized probability estimates, costs per option, explicit elicitation of values, information about others’ opinions, coaching on decision theory concepts, personalized recommendations, a formal decision analysis, or other components.
4. **IF** this study is about patients, is there at least one Decision Support Tool/Decision Aid mentioned, **OR**, **IF** the study is about providers, is there an intervention to increase the use of a DST or increase shared decision‐making?

#### Omega3

**Table:**

**Patient types** We are interested in the following patient types 1. Patients who are healthy 2. Patients who have diabetes, metabolic syndrome or hypertension 3. Patients who have dyslipidemia (high cholesterol or triglycerides) 4. Patients with existing or previous cardiovascular/heart disease, including heart attack (myocardial infarction), atherosclerosis, stroke, arrhythmia, heart failure, etc. 5. Patients with symptoms of any of the above mentioned conditions **EXCLUDE:** Patients selected for having a non‐cardiovascular disease, **OR**, a non‐diabetes related disease *(*e.g. *cancer, gastrointestinal disease, dialysis, chronic renal failure, rheumatic disease)*, **OR**, a condition *(*e.g. *pregnancy)*. **Primary study on humans** A study reporting data from an experiment or from observations on a group of human beings (i.e. not just human cells, eyes etc.). Examples of things that are not primary studies, even if describing observations on a group of human beings: *Reviews, editorials, guidelines.*

1. Does the abstract imply it describes a primary study with adult patients (>= 18 years old), who fit the patient types described above?
  **Table:**
  We are interested in the effects of omega‐3 fatty acids. Synonyms of “omega‐3 fatty acids” include “n‐3 fatty acids”, “(long chain) PUFA”, “(long chain) polyunsuturated fatty acids”. **Supplements, diet plans and foods that contain omega‐3 fatty acids** 1. Fish oil (*Incl. menhaden oil, sea mammals, marine, seaweed*) 2. ALA oils (*Incl. flax seed, linseed, walnut, butternut, pumpkin seed, canola/rapeseed, soy, wheatgerm mustard seed*) 3. n‐3 components (*Incl. EPA eicosapentaenoic acid, DHA docosahexaenoic acid, ALA alpha‐linolenic acid, DPA docosapentaenoic acid, SDA stearidonic acid*) 4. Fish‐rich diets or Mediterranean diets, of “Food frequency Questionnaires” (FFQs) (***ONLY IF*** *the paper describes the average daily amount of omega‐3 rich foods consumed, e.g., 300g of fatty fish/week, or 250 g of walnuts per week; or translates food intake to the corresponding omega‐3 fatty acid intake*)
2. Are patients receiving any of the above mentioned supplements, diet plans, or fortified foods?

#### Outcome & Study type table

Looking at the table below, each column describes a factor to look for (e.g. the study type) and the entry in each row describes which values are acceptable for that factor, assuming the other factors were acceptable in the same row. E.g., If an abstract described a Cardiovascular disease outcome, had a follow‐up duration of 2 years and was conducted as a RCT (*Randomized controlled trial*), you would answer *Yes*, since you could answer *Yes* to all factors in row 1 of the table.

**Table:**

Accepted outcome types	Minimum follow‐up duration	Minimum number of participants	Accepted study types
Cardiovascular disease outcomes Blood pressure or lipid outcomes Adverse events (*only from intervention studies of supplements*)	1 year 4 weeks any	none none 100	We are interested in studies that: 1. Allow a comparison between different levels of omega‐3 fatty acid intake, e.g., fish oil vs no fish oil or placebo, or fewer fish/week vs more fish/week 2. Follow‐up people over time **Examples of eligible studies** **Randomized controlled trial RCT** A study in which a number of similar people are randomly assigned to 2 (or more) groups to test a specific drug or treatment. One group (the experimental group) receives the treatment being tested, the other (the comparison or control group) receives an alternative treatment, a dummy treatment (placebo) or no treatment at all. The groups are followed up to see how effective the experimental treatment was. (Source: http://en.wikipedia.org/wiki/Randomized_controlled_trial) **Non‐randomized comparative trial** An experimental study in which people are allocated to different interventions using methods that are not random. (Source: https://ccg.cochrane.org/non‐randomised‐controlled‐study‐nrs‐designs) **Retrospective or prospective cohort** In a retrospective cohort study, the medical records of groups of individuals who are alike in many ways but differ by a certain characteristic are compared for a particular outcome (Source: http://en.wikipedia.org/wiki/Retrospective_cohort_study) A prospective cohort study is a cohort study that follows over time a group of similar individuals (cohorts) who differ with respect to certain factors under study, to determine how these factors affect rates of a certain outcome (Source: http://en.wikipedia.org/wiki/Prospective_cohort_study) **Nested case‐control study** A nested case‐control study uses data previously collected from a large cohort study to compare a subset of participants without the outcome with participants who developed the outcome. (Source: http://en.wikipedia.org/wiki/Nested_case‐control_study) **Examples of non‐eligible studies** **Case‐control study** A case‐control study is a type of observational study in which two existing groups differing in outcome are identified and compared on the basis of some supposed causal attribute. (Source: http://en.wikipedia.org/wiki/Case‐control_study) **Cross‐sectional study** A cross‐sectional study (also known as a cross‐sectional analysis, transversal study, prevalence study) is a type of observational study that involves the analysis of data collected from a population, or a representative subset, at one specific point in time. (Source: http://en.wikipedia.org/wiki/Cross‐sectional_study)

**Cardiovascular disease outcomes**

- CVD‐related (myocardial infarction, stroke) death
- non‐fatal CVD events (myocardial infarction, acute coronary syndrome, stroke/CVA, TIA, unstable angina, amputation 2° PVD)
- coronary/cardiac disease
- peripheral vascular disease (PVD)
- congestive heart failure (CHF)
- pulmonary edema
- ventricular arrhythmia (tachycardia, tachyarrhythmia, fibrillation, bradycardia)
- sudden death
- atrial fibrillation
- supraventricular tachycardia
- cardiovascular invasive interventions (revascularization, CABG (bypass), PCI (coronary angioplasty), vascular (arterial) surgery (carotid, peripheral), Thrombolysis (eg, tPA to dissolve clot))

**Blood pressure or lipid outcomes**

- blood pressure (new‐onset hypertension, SBP, DBP, MAP)
- Key lipid values (HDL‐cholesterol, LDL‐cholesterol, triglycerides, LDL:HDL, TC:HDL)
- Can you answer 'Yes' or 'I Cannot Tell' to all 4 columns in a row, in the outcome and study type table above?

## APPENDIX C—ANONYMIZED WORKER SATISFACTION RESPONSES, REVIEWS, ETC

### C—ANONYMIZED WORKER SATISFACTION RESPONSES, REVIEWS, ETC.1

The following statements are a selection of real statements provided by workers either via email, through the Mechanical Turk messaging system or on the review site Turkopticon (https://turkopticon.ucsd.edu/ADWWO1HSTPYS5).
I have been doing quite a few of your HITs recently (Worker ID: XXX), and I would like to say you are a truly outstanding requester. I genuinely appreciate your feedback with control questions, and I hope I have provided useful data for your research. **Andrew, Mechanical Turk worker**
You have some of the most engaging hits on MTurk. **Phillip, Mechanical Turk worker**
This is a genuinely phenomenal requester. I've done over 500 HITs without a rejection‐ they pay and approve promptly. When you are doing a task incorrectly they implement control questions and give you feedback on how to improve your work‐ something I GREATLY appreciate. **Anonymous worker on Turkopticon**
So happy I stumbled on this requester. The HITs are very engaging for me **Anonymous worker on Turkopticon**
I would recommend these if you are interested in science and are willing to learn a bit. Definitely do not attempt these if you want to do something flippantly, these require concentration and critical thinking. **Anonymous worker on Turkopticon**

## APPENDIX D—HONEYPOT DETAILS

### D—HONEYPOT DETAILS.1

This Appendix details the acceptance, rejection, and injection of our hidden control tests, commonly referred to as *honeypots*.

#### Injecting honeypots into HITs

Whenever a worker accessed an HIT, there was a chance that 1 of the 3 loaded citations was a honeypot, ie, a citation for which the system knew both all the correct answers and why these were the correct answers. If the worker was unknown to us, there was a 30% chance of a honeypot. If the worker had been subjected to at least 3 honeypots, there was a 20% chance. Finally, if the worker had been subjected to at least 6 honeypots, there was a 10% chance, up until the worker had been subjected to all 10 honeypots.

#### Accepting/rejecting honeypots

When a worker submitted an HIT with a honeypot loaded as 1 of the 3 citations, the answers for that honeypot was sent to our server using a JavaScript callback (via AJAX) before sending the full data to Mechanical Turk. Our server then determined whether the answers were correct and if not, to which degree they were incorrect.

If the answers were correct for all questions, an “OK” message was sent back to the frontend and the HIT submitted without the worker being aware of the presence of a honeypot. If the answers were not correct, a per‐question response was generated for the worker, such that each wrong answer was shown along with the right answer and the reason for it being the right answer. These explanations were written before the experiment and stored with the honeypot answers. Upon completion, the generated response would be presented to the worker, asking to confirm by pressing an “OK” button, which then submitted the answers to Mechanical Turk as is.

For honeypot responses to workers we distinguished between 2 situations: corrections and failures. A correction was when the worker had less than 50% of the answers incorrect, ie, such that the answers would not sway a majority decision strategy. In such a case, the error response to the worker was displayed, but the worker had not failed the honeypot and was informed of that fact. A failure on the other hand was when at least 50% of answers were wrong. In that case the error response was shown to the worker and the worker was registered as having failed the honeypot.

If any worker failed more than 1/3 of all honeypots, counting from having finished at least 3, the worker was blocked from any future work on that set of HITs.
